# Expression of genes controlling fat deposition in two genetically diverse beef cattle breeds fed high or low silage diets

**DOI:** 10.1186/1746-6148-9-118

**Published:** 2013-06-17

**Authors:** Ana Sofia Henriques da Costa, Virgínia Maria Rico Pires, Carlos Mendes Godinho Andrade Fontes, José António Mestre Prates

**Affiliations:** 1 Secção de Bioquímica, CIISA, Faculdade de Medicina Veterinária, Universidade Técnica de Lisboa, Av. da Universidade Técnica, Pólo Universitário do Alto da Ajuda, Lisboa 1300-477, Portugal

**Keywords:** Beef cattle, Adipose tissue, Muscle, Gene expression, Fat deposition

## Abstract

**Background:**

Both genetic background and finishing system can alter fat deposition, thus indicating their influence on adipogenic and lipogenic factors. However, the molecular mechanisms underlying fat deposition and fatty acid composition in beef cattle are not fully understood. This study aimed to assess the effect of breed and dietary silage level on the expression patterns of key genes controlling lipid metabolism in subcutaneous adipose tissue (SAT) and *longissimus lumborum* (LL) muscle of cattle. To that purpose, forty bulls from two genetically diverse Portuguese bovine breeds with distinct maturity rates, Alentejana and Barrosã, were selected and fed either low (30% maize silage/70% concentrate) or high silage (70% maize silage/30% concentrate) diets.

**Results:**

The results suggested that enhanced deposition of fatty acids in the SAT from Barrosã bulls, when compared to Alentejana, could be due to higher expression levels of lipogenesis (*SCD* and *LPL*) and β-oxidation (*CRAT*) related genes. Our results also indicated that *SREBF1* expression in the SAT is increased by feeding the low silage diet. Together, these results point out to a higher lipid turnover in the SAT of Barrosã bulls when compared to Alentejana. In turn, lipid deposition in the LL muscle is related to the expression of adipogenic (*PPARG* and *FABP4*) and lipogenic (*ACACA* and *SCD*) genes. The positive correlation between *ACACA* expression levels and total lipids, as well trans fatty acids, points to ACACA as a major player in intramuscular deposition in ruminants. Moreover, results reinforce the role of *FABP4* in intramuscular fat development and the SAT as the major site for lipid metabolism in ruminants.

**Conclusions:**

Overall, the results showed that SAT and LL muscle fatty acid composition are mostly dependent on the genetic background. In addition, dietary silage level impacted on muscle lipid metabolism to a greater extent than on that of SAT, as evaluated by gene expression levels of adipogenic and lipogenic factors. Moreover, the response to diet composition evaluated through mRNA levels and fatty acid composition showed interesting differences between Alentejana and Barrosã bulls. These findings provide evidence that the genetic background should be taken into account while devising diet-based strategies to manipulate fatty acid composition of beef cattle tissues.

## Background

During the last decades consumers have started demanding animal products of low fat and high polyunsaturated fatty acids content, while maintaining high and consistent quality [1,2]. To that purpose, research has been conducted on ruminant’s adipogenesis and lipogenesis in order to improve both the production efficiency and beef quality. Adipose tissue is involved in the regulation of body homeostasis, particularly in energy metabolism, storage and expenditure. In cattle, fatty acid type and amount in muscles are directly associated with meat quality and its value. In fact, subcutaneous and intramuscular adipose tissues are the most important fat depots concerning meat quality traits. It is desirable that cattle carcasses have minimal amounts of fat stored in subcutaneous adipose tissue (SAT), without a detrimental decrease in intramuscular fat [3]. This can be achieved only if the regulation of lipid deposition in intramuscular and other fat depots differs substantially. Despite being the main site for *de novo* fatty acid and triacylglycerols (TAG) synthesis in ruminants [4], the SAT is also the most energetically inefficient fat depot and, therefore, considered an economic loss. However, while during the last decade knowledge of rodents and human fat physiology has progressed rapidly [5], the same information regarding ruminant species is very limited.

The expression level of adipogenic and lipogenic genes in adipose tissues is regulated by a number of transcription factors [6], whose differential expression is known to play a key role in lipid metabolism of cattle adipocytes [7]. Adipogenesis, lipogenesis, and lipolysis occur through the interaction of endogenous genetic mechanisms (mediated through gene expression and regulated by intrinsic factors), external controls (endocrine agents, extrinsic factors and nutritional metabolites), as well as local interactions within cells in a fat depot [8]. Despite the intricacies of lipogenesis and lipolysis, the role of some genes has been elucidated and confirmed to be related to fatty acid composition in cattle [6]. Potential regulatory mechanisms involved in the fatty acid deposition are lipogenic (*ACACA*, *LPL*, *FABP4* and *SCD*) and oxidative (*CPT1B* and *CRAT*) genes, as well as transcription regulators (*PPARA*, *PPARG* and *SREBF1*). These genes have been described [9-11] for their roles and expression patterns during adipocyte differentiation, namely in studies comparing the regulation of adipose tissue deposition in distinct cattle breeds [9,12].

Genetic factors underlying both the deposition and the turnover of individual fatty acids are not fully understood, although breed has been found to influence beef fatty acid composition [13]. Fatty acid composition in meat-producing animals is recognised to have implications on the nutritional and organoleptic properties of meat, as well as in its technological quality [14]. Ruminant products can be an additional source of the beneficial long-chain *n*-3 PUFA (EPA and DHA) for human diets [2] when the consumption of *n*-3 PUFA-rich foods, such as fish, is low. In addition, ruminant meats are major dietary sources of DPA (22:5*n*-3). Thus, genetic selection and breeding of animals with a desirable meat fatty composition may provide a source of beneficial fatty acids for human consumption [15]. Comparative differences of beef cattle present a unique resource to study several aspects of lipid metabolism. In addition to genetic factors, finishing systems can dramatically alter fat deposition [16], thus indicating that lipogenic activity is influenced by the dietary energy level, the energy source and, possibly, the forage to concentrate ratio. However, fatty acid composition in ruminant animals, unlike in monogastrics, is much less dependent on the diet, as a consequence of dietary fatty acid metabolism (i.e., isomerisation, biohydrogenation) within the rumen [17].

The biochemical processes and the molecular background affecting genetic variability of the complex trait of fat content and fatty acid composition are not yet fully understood, particularly with regard to European cattle breeds, because most of the recent studies have been performed on the specific genetic background of Japanese Black cattle [15]. Therefore, we conducted a trial with bulls from two Portuguese genetically diverse breeds [18] with distinct maturity rates, Alentejana and Barrosã, fed high or low silage diets. The working hypothesis of the present paper was that the expression of key genes controlling lipid metabolism during the finishing phase of cattle is breed-specific (Alentejana *vs*. Barrosã) and diet-modulated (high *vs*. low silage). Furthermore, given the distinct roles of subcutaneous and intramuscular fat depots in cattle lipid metabolism, the tissue-specific variations were also investigated through the analysis of both SAT and muscle gene expression patterns. Finally, the breed-, diet- and tissue-specific relationships among the expression level of these genes and fat content and composition were also assessed.

## Results

### Fatty acid composition of subcutaneous adipose tissue

Total lipid content and percentages of main fatty acids (*i.e*., those >1% of total fatty acids and the main CLA isomer, 18:2*c*9*t*11) in the SAT from the four experimental groups are presented in Table 1. The content of total lipids was higher (*P* = 0.002) in the SAT of Barrosã breed when compared to Alentejana bulls. Barrosã bulls had lower 14:0 (*P* = 0.024), 16:0 (*P* < 0.001) and 18:0 (*P* < 0.001) fatty acid proportions in the SAT than the Alentejana bulls. Breed also determined the proportions of 16:1*c*9, *a*-17:0, 18:1*t*11, 18:1*c*9 and 18:2*c*9*t*11-CLA fatty acids in the SAT, with the Barrosã breed having higher values when compared to the Alentejana bulls (*P* < 0.01). *Trans* fatty acids (TFA) were higher in Barrosã than in Alentejana bulls (*P* = 0.004). Diet influenced the proportions of *a*-17:0 and 18:0 (*P* < 0.001 and *P* = 0.034, respectively), with the high silage fed bulls presenting the highest values. Animals fed the low silage diet had the highest proportions of TFA (*P* = 0.037). The branched chain fatty acids (BCFA), which are closely related to the rumen activity, were higher in animals fed the high silage diets (*P* < 0.001). A breed × diet interaction was found for the fatty acid 18:2*n*-6 (*P* = 0.002), with the Alentejana bulls fed the low silage diet presenting the highest proportion. While Alentejana bulls had higher proportions of saturated (SFA) but lower monounsaturated fatty acids (MUFA) percentages, the inverse pattern was observed in the Barrosã bulls (*P* < 0.001). Both total PUFA and *n*-6 PUFA percentages were higher in the Alentejana bulls fed the low silage diet, whereas in the SAT from Barrosã bulls no variation between diets was observed (breed × diet, *P* = 0.001). The percentages of *n*-3 PUFA were the highest in the SAT from Barrosã bulls fed the high silage diet (*P* = 0.012).

**Table 1:** **Total lipids and fatty acid composition of subcutaneous adipose tissue from Alentejana and Barrosã breeds fed either high or low silage-based diets**^**1-3**^

	**Alentejana**				**Barrosã**				***P***		
	**HS**		**LS**		**HS**		**LS**		**Breed**	**Diet**	**Breed × Diet**
	**Mean**	**SE**	**Mean**	**SE**	**Mean**	**SE**	**Mean**	**SE**			
Total lipids	58.44	1.748	59.67	1.063	63.89	1.729	65.17	1.689	0.002	0.434	0.987
*Main individual fatty acids*											
14:0^§^	3.50	0.198	3.52	0.189	2.98	0.130	3.23	0.164	0.024	0.426	0.517
16:0^§^	27.24	0.429	26.57	0.552	23.63	0.444	25.11	0.659	<0.001	0.449	0.051
16:1*c*9^§^	4.03	0.380	3.83	0.242	4.89	0.263	5.41	0.248	<0.001	0.590	0.218
*a*-17:0^§^	1.30	0.026	1.13	0.031	1.45	0.035	1.35	0.038	<0.001	<0.001	0.315
18:0	14.53	0.776	12.94	0.804	11.44	0.713	9.75	0.658	<0.001	0.034	0.946
18:1*t*11	1.41	0.123	1.36	0.129	1.88	0.125	1.74	0.113	0.002	0.456	0.705
18:1*c*9^§^	33.5	1.019	34.65	0.835	37.43	0.752	37.18	0.872	<0.001	0.610	0.431
18:1*c*11	4.13	0.396	3.7	0.367	4.32	0.144	4.53	0.142	0.093	0.692	0.279
CLA(*c*9*t*11)	0.40	0.034	0.34	0.026	0.80	0.041	0.81	0.041	<0.001	0.504	0.324
18:2*n*-6	1.49^b^	0.030	2.29^c^	0.141	1.70^a^	0.050	1.82^a^	0.064	0.181	<0.001	0.002
*Partial sums*											
Σ SFA	46.89	1.000	44.76	1.303	39.61	1.035	39.21	1.184	<0.001	0.273	0.453
Σ MUFA	44.89	1.034	46.00	1.323	50.62	1.092	51.54	1.223	<0.001	0.395	0.933
Σ TFA	2.73	0.181	3.57	0.277	3.75	0.176	3.79	0.145	0.004	0.037	0.057
Σ PUFA	1.80^a^	0.101	2.58^c^	0.144	2.07^b^	0.055	2.11^b^	0.067	0.347	<0.001	0.001
Σ *n*-3 PUFA	0.27^a^	0.012	0.26^a^	0.016	0.32^b^	0.008	0.25^a^	0.005	0.081	<0.001	0.012
Σ *n*-6 PUFA	1.53^a^	0.090	2.32^c^	0.134	1.75^b^	0.049	1.86^b^	0.065	0.216	<0.001	0.001
Σ BCFA	2.69	0.085	2.06	0.051	2.77	0.068	2.25	0.060	0.051	<0.001	0.411

^1^Different superscripts differ at least *P* < 0.05. *SFA*, sum of 12:0,14:0, 15:0, 16:0, 17:0, 18:0 and 20:0; *MUFA*, sum of 14:1c9, 16:1c7, 16:1c9, 17:1c9, 18:1c9, 18:1c11, 18:1c12, 18:1c13, 18:1c15, 19:1 and 20:1c11; *TFA*, sum of 18:1t6-t8, 18:1t9, 18:1t10, 18:1t11, 18:1t12, 18:1t16 + c14, 18:2c9t11 and 18:2t11c15; *PUFA*, sum of 18:2n-6, 18:3n-3, 20:3n-6 and 20:4n-6; *BCFA*, sum of i-14:0, i-15:0, a-15:0, i-16:0, i-17:0, a-17:0 and i-18:0.

^2^Total lipids are expressed as g/100 g muscle; fatty acid composition is expressed as g/100 g fatty acids.

^3^*HS*, High silage; *LS*, Low silage.

^§^Variable adjusted for total lipids.

### Fatty acid composition of muscle

The values for total lipids and main fatty acids (*i.e*., those >1% of total fatty acids and 18:2*c*9*t*11) in the muscle from the four experimental groups are presented in Table 2. Total lipid contents were higher in the muscle from Barrosã bulls fed the low silage diet in comparison to those fed the high silage diet, whereas no variation was observed for the Alentejana bulls (breed × diet, *P* = 0.001). The 14:0 proportion in the muscle was higher (*P* < 0.001) in the Barrosã breed when compared to the Alentejana bulls. Breed also influenced the percentages of 16:1*c*9, 18:1*c*9 and 18:2*c*9*t*11-CLA in the muscle, with the Barrosã having higher values when compared to the Alentejana breed (*P* < 0.001). Barrosã bulls fed the low silage diet had (*P* = 0.037) the highest proportions of SFA. Both MUFA and TFA proportions were higher (*P* < 0.001) in Barrosã when compared to Alentejana bulls. Alentejana bulls had the highest percentages of total, *n*-3 and *n*-6 PUFA (*P* < 0.001). The 14:0 percentages were higher (*P* = 0.003) in the low silage than in the high silage fed bulls. Barrosã bulls fed the low silage diet had the highest 16:0 fatty acid proportions (breed × diet, *P* = 0.003). Deposition of 20:4*n*-6 varied according to breed and diet (*P* < 0.001 and *P* = 0.001, respectively), with Alentejana having higher percentages than Barrosã bulls, and low silage promoting lower deposition in comparison to high silage diet. Feeding the low silage diet resulted in higher percentages (*P* = 0.038) of TFA in comparison to the high silage diet. The BCFA were higher (*P* < 0.001) in animals fed on the high silage diet. Bulls fed the high silage diet had higher *n*-3 PUFA percentages (*P* < 0.001) than those fed the low silage diet. The Barrosã bulls fed the low silage diet had the lowest percentages of 18:2*n*-6 (breed × diet, *P* = 0.007). The deposition of 18:0 was similar (*P* > 0.05) across the four experimental groups.

**Table 2:** **Total lipids and fatty acid composition of *longissimus lumborum *muscle from Alentejana and Barrosã breeds fed either high or low silage diets**^**1-3**^

	**Alentejana**				**Barrosã**				***P***		
	**HS**		**LS**		**HS**		**LS**		**Breed**	**Diet**	**Breed × Diet**
	**Mean**	**SE**	**Mean**	**SE**	**Mean**	**SE**	**Mean**	**SE**			
Total lipids^§^	1.21^a^	0.758	1.25^a^	0.836	1.76^b^	1.208	2.76^c^	1.955	<0.001	<0.001	0.001
*Main individual fatty acids*											
14:0^†^	2.04	0.082	2.24	0.121	2.29	0.075	2.71	0.098	<0.001	0.003	0.261
16:0^†^	23.50^a^	0.362	23.17^a^	0.439	23.33^a^	0.325	25.40^b^	0.373	0.011	0.028	0.003
16:1*c*9	2.37	0.162	2.52	0.102	2.85	0.103	3.27	0.189	<0.001	0.057	0.361
18:0	15.14	0.709	14.04	0.492	14.20	0.424	14.04	0.423	0.378	0.244	0.380
18:1*c*9^‡^	28.36	0.702	28.85	0.783	31.04	0.823	33.52	0.645	<0.001	0.053	0.189
18:1*c*11	2.90	0.302	3.35	0.083	3.40	0.254	3.08	0.088	0.586	0.762	0.078
CLA(*c*9*t*11)	0.21	0.011	0.20	0.014	0.45	0.025	0.45	0.018	<0.001	0.815	0.789
18:2*n*-6^*^	7.39^c^	0.387	8.32^c^	0.719	6.07^b^	0.329	4.22^a^	0.251	<0.001	0.333	0.007
20:4*n*-6^†^	2.39	0.156	2.25	0.148	1.53	0.140	1.28	0.117	<0.001	0.001	0.052
*Partial sums*											
Σ SFA^†^	42.10^a^	0.755	40.69^a^	0.815	41.29^a^	0.672	43.22^b^	0.624	0.209	0.736	0.037
Σ MUFA^‡^	35.77	0.876	36.92	0.866	41.58	0.825	40.31	0.939	<0.001	0.952	0.206
Σ TFA	1.65	0.095	2.03	0.145	2.49	0.106	2.62	0.114	<0.001	0.038	0.294
Σ PUFA^*^	11.92	0.465	12.57	0.929	9.11	0.959	7.29	0.491	<0.001	0.446	0.111
Σ *n*-3 PUFA^†^	1.50	0.095	1.13	0.059	1.23	0.134	0.661	0.038	<0.001	<0.001	0.288
Σ *n*-6 PUFA^*^	10.27	0.407	11.34	0.886	7.90	0.791	6.55	0.472	<0.001	0.843	0.083
Σ BCFA	1.66	0.062	1.41	0.057	1.71	0.054	1.46	0.054	0.394	<0.001	0.956

^§^Published in [19].

^1^Different superscripts differ at least *P* < 0.05. *SFA*, sum of 12:0, 14:0, 15:0, 16:0, 17:0, 18:0, 20:0 and 21:0; *MUFA*, sum of *c*9-14:1, *c*7-16:1, *c*9-16:1, *c*9-17:1, *c*9-18:1, *c*11-18:1, *c*12-18:1, *c*13-18:1, 18:1*c*15 and *c*11-20:1; *TFA*, sum of 18:1t*6*-t8, 18:1t9, 18:1t10, 18:1t11, 18:1 t16 + c14 and 18:c9t11; *PUFA*, sum of 18:2n-6, 18:3n-6, 18:3n-3, 20:2n-6, 20:3n-6, 20:3n-9, 20:4n-6, 20:5n-3, 22:4n-6, 22:5n-3 and 22:6n-3; *BCFA*, sum of *i*-15:0, *a*-15:0, *i*-16:0, *i*-17:0, *a*-17:0 and *i*-18:0.

^2^Total lipids are expressed as g/100 g muscle; fatty acid composition is expressed as g/100 g fatty acids.

^3^*HS* high silage, *LS* low silage.

^†^Variable adjusted for breed × total lipids; ^‡^ Variable adjusted for total lipids; ^*^ Variable adjusted for breed × diet × total lipids.

### Gene expression in subcutaneous adipose tissue

**Figure 1:**
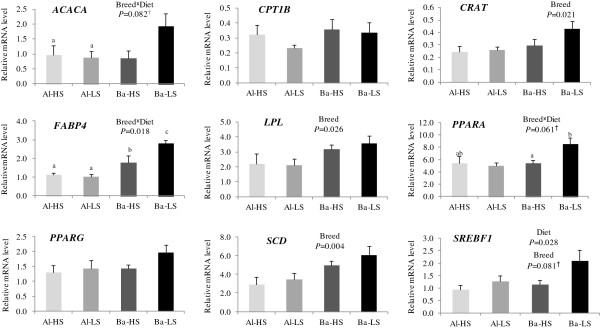
**Relative expression levels of nine genes in the subcutaneous adipose tissue of Alentejana and Barrosã bulls fed high or low silage diets. **Each value was normalized to *RPLP0 *expression. Al-HS: Alentejana bulls fed the high silage diet; Al-LS: Alentejana bulls fed the low silage diet; Ba-HS: Barrosã bulls fed the high silage diet; Ba-LS: Barrosã bulls fed the low silage diet. Error bars indicate standard error. *FABP4 *relative expression levels were adjusted for SAT total lipids content. ^†^Tendencies were considered for 0.05 < *P* < 0.10. ^a,b,c ^Least square means with different superscripts differ at least *P* < 0.05.

The relative mRNA expression levels of the nine lipid metabolism key factors analysed in the SAT are presented in Figure 1. *CRAT*, *LPL* and *SCD* showed higher expression levels in the Barrosã breed when compared to the Alentejana animals (*P* < 0.05), corresponding to a fold-change of 1.46, 1.56 and 1.74, respectively. In addition, the Barrosã breed tended (*P* = 0.081) to have higher expression levels (1.47-fold) of SREBF1 than the Alentejana bulls. The low silage diet promoted the *SREBF1* up-regulation (1.64-fold) in comparison to the high silage diet (*P* = 0.028). Concerning the *ACACA* mRNA levels, while the Barrosã bulls fed the low silage diet tended to have higher expression levels than the high silage fed ones (2.24-fold), no variation between dietary treatments were found for the Alentejana bulls (breed × diet, *P* = 0.082). A similar pattern was observed for the *PPARA* gene expression levels (1.6-fold, breed × diet, *P* = 0.061). As for the *FABP4* gene expression, higher expression levels were found in the low silage fed Barrosã bulls when compared to those fed the high silage diet (1.55-fold), whilst in the Alentejana bulls there were no variations between feeding regimens (breed × diet, *P* = 0.022).

### Gene expression in muscle

**Figure 2:**
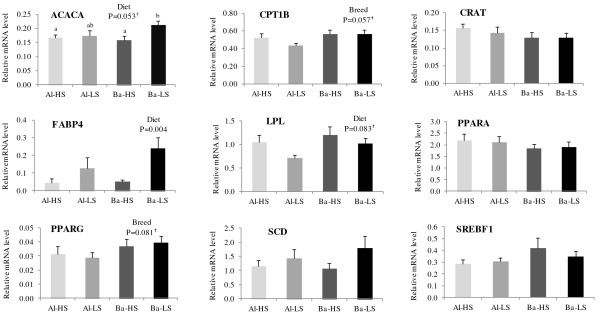
**Relative expression levels of nine genes in the *longissimus lumborum *muscle of Alentejana and Barrosã fed high or low silage diets. **Each value was normalized to *PPIB *expression. Al-HS: Alentejana bulls fed the high silage diet; Al-LS: Alentejana bulls fed the low silage diet; Ba-HS: Barrosã bulls fed the high silage diet; Ba-LS: Barrosã bulls fed the low silage diet. *ACACA* and *PPARG *relative expression levels were adjusted for total lipids content. *CRAT *relative expression levels were adjusted for breed × diet × total lipids content. *FABP4* relative expression levels were adjusted for diet × total lipids content. *SCD *relative expression levels were adjusted to breed × total lipids content. Error bars indicate standard error. ^†^Tendencies were considered for 0.05 < *P* < 0.10. ^a,b,c ^Least square means with different superscripts differ at least P < 0.05.

The relative mRNA expression levels of the nine lipid metabolism key factors analysed in the LL muscle are presented in Figure 2. The CPT1B encoding gene tended to have higher expression levels (1.18-fold) in the Barrosã breed when compared to the Alentejana bulls (*P* = 0.057). The *PPARG* gene showed a similar tendency (1.28-fold, *P* = 0.081). The *ACACA* mRNA levels tended to be 1.19-fold higher in the low silage diet fed bulls when compared to the high silage fed ones (*P* = 0.053). Conversely, the mRNA levels of *LPL* tended (*P* = 0.083) to be higher (1.29-fold) in the high silage fed bulls than in their low silage fed counterparts. The *FABP4* gene showed higher expression levels (3.83-fold) in the low silage fed when compared to the high silage fed bulls (*P* = 0.007), corresponding to a fold-change of 3.83. The expression levels of *CRAT*, *PPARA*, *SCD* and *SREBF1* genes were similar across experimental groups (*P* > 0.05).

### Correlation between gene expression and fatty acids

In order to elucidate the possible contribution of the lipogenic enzymes for fatty acid composition in SAT and muscle, the relationship between levels of gene expression and the content on main fatty acids was determined (Table 3).

**Table 3:** **Pearson’s correlation coefficients between genes expression levels and fatty acid composition in subcutaneous adipose tissue and *longissimus lumborum *muscle from Alentejana and Barrosã breeds**^**1-2**^

	**TL**	**14:0**	**16:0**	**18:0**	**18:1 *t *11**	**18:1 *c *9**	**18:1 *c *11**	**CLA**	**SFA**	**MUFA**	**TFA**
**SAT**											
*ACACA*	0.22	−0.10	−0.16	−0.11	0.09	0.26	−0.1	0.25	−0.17	0.19	0.06
*CPT1B*	−0.02	−0.04	−0.09	0.20	−0.04	−0.06	0.08	0.04	0.07	−0.05	−0.14
*CRAT*	0.21	−0.13	−0.25	−0.12	0.12	0.27	−0.15	0.26	−0.22	0.20	0.10
*FABP4*	0.38*	−0.22	−0.45**	−0.41*	0.27	0.34*	0.37*	0.58***	0.51***	0.48**	0.28
*LPL*	0.34*	−0.33*	−0.42**	−0.16	0.15	0.48**	−0.08	0.35*	−0.34*	0.35*	0.07
*PPARA*	0.21	−0.10	−0.18	−0.14	−0.03	0.28	−0.11	0.20	−0.19	0.23	−0.05
*PPARG*	0.14	−0.20	−0.13	−0.12	−0.04	0.25	0.02	0.10	−0.18	0.21	0.02
*SCD*	0.24	−0.31	−0.48**	−0.26	0.13	0.51**	−0.03	0.45**	−0.44**	0.43**	0.15
*SREBF1*	0.15	−0.19	−0.16	−0.19	−0.01	0.26	0.00	0.19	−0.23	0.24	0.08
**Muscle**											
*ACACA*	0.48**	0.15	0.20	0.01	0.25	0.20	0.10	0.14	0.20	0.23	0.40*
*CPT1B*	0.13	−0.05	−0.06	0.00	0.18	0.14	0.28	0.20	−0.11	0.21	0.05
*CRAT*	−0.17	−0.18	−0.13	0.20	−0.16	−0.11	0.17	−0.30	0.05	−0.10	−0.33*
*FABP4*	0.36*	0.19	0.20	−0.16	0.26	0.42**	−0.11	0.17	0.07	0.38*	0.21
*LPL*	0.09	−0.03	−0.05	−0.21	0.02	0.27	−0.06	0.14	−0.21	0.25	−0.13
*PPARA*	−0.19	0.08	0.17	−0.06	−0.30	−0.33*	0.01	−0.21	0.09	−0.30	−0.27
*PPARG*	0.21	0.35*	0.28	−0.27	0.17	0.22	−0.18	0.30	0.03	0.23	0.09
*SCD*	0.25	−0.05	0.04	0.08	0.26	0.13	0.14	0.13	0.19	0.12	0.39*
*SREBF1*	0.08	0.14	0.16	−0.01	0.05	−0.14	0.48**	0.31	0.13	0.03	0.33*

^1^*TL*, total lipids; *SAT*, subcutaneous adipose tissue.

^2^Significance level *, *P* < 0.05; **, *P* < 0.01; ***, *P* < 0.001.

For SAT, the correlation analysis revealed a positive relationship between total lipid content and *FABP4* gene expression level (*r* = 0.38). In addition, this analysis showed negative correlations between this gene and 16:0 and 18:0 fatty acids (*r* = 0.45 and *r* = 0.41, respectively). Furthermore, positive correlations were found for *FABP4* expression level and 18:1*c*9 (*r* = 0.34), 18:1*c*11 (*r* = 0.37) and *c*9,*t*11-CLA (*r* = 0.58), as well as for SFA (*r* = 0.51) and MUFA (*r* = 0.48). *LPL* gene expression had a negative association with 14:0 (*r* = −0.33) and 16:0 (*r* = −0.42), as well as the SFA (*r* = −0.34). In addition, positive correlations were found between *LPL* mRNA expression levels and total lipids (*r* = 0.34), 18:1*c*9 (*r* = 0.48) and *c*9,*t*11-CLA (*r* = 0.35), as well as with MUFA (*r* = 0.35). The *SCD* gene expression and the proportions of both 18:1*c*9 and *c*9,*t*11-CLA were positively correlated (*r* = 0.51 and *r* = 0.45, respectively). A moderate association was also observed between *SCD* gene expression and MUFA (*r* = 0.43). In addition, there was a negative correlation between 16:0 and expression of the *SCD* gene (*r* = −0.48). Concomitantly, SFA and *SCD* gene expression were also negatively correlated (*r* = −0.44).

Concerning the muscle, *ACACA* expression levels were correlated with total lipids (*r* = 0.48), and the percentages of TFA (*r* = 0.40). Correlations were also found between *FABP4* mRNA levels and total lipids (*r* = 0.36), 18:1*c*9 (*r* = 0.42), and MUFA (*r* = 0.38). The mRNA levels for *PPARA* gene were negatively related to 18:1*c*9 (*r* = −0.33) percentages. Levels of *PPARG* expressionwere associated with the percentages of 14:0 *(r* = 0.35). A positive association was found for *SCD* mRNA levels and TFA (*r* = 0.39). Finally, 18:1*c*11 and TFA showed positive correlations (*r* = 0.48 and *r* = 0.33, respectively) with the expression levels of the *SREBF1* gene.

### Principal components analysis

**Figure 3:**
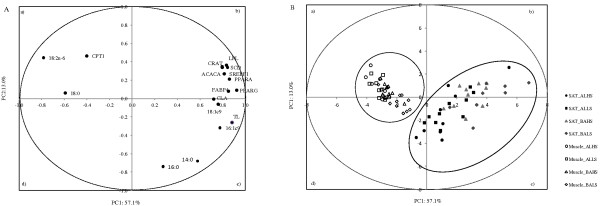
**Loading plot of the first and second principal components (PC) of the pooled data (A) and component’s score vectors (B) for *longissimus lumborum *muscle and subcutaneous adipose tissue from Alentejana and Barrosã bulls fed high or low silage diets. **TL: total lipids; SAT: subcutaneous adipose tissue; Al-HS: Alentejana bulls fed the high silage diet; Al-LS: Alentejana bulls fed the low silage diet; Ba-HS: Barrosã bulls fed the high silage diet; Ba-LS: Barrosã bulls fed the low silage diet.

A Principal Components Analysis (PCA) was applied to a data set of fatty acid composition and gene expression parameters in order to describe the variability of the pooled data into two dimensions (Figure 3A). The score plot of the first two components explains 70.1% of the total variability, with 57.1% for PC1 and 13.0% for PC2 (Table 4). In the score plot, there is a clear separation of most genes from the main fatty acids, except 18:1c9 and CLA (*c*9*t*11)*.* Most of the genes were allocated on the right side of the plot (quadrant *b*), clearly separated from the other variables, showing positive scores for the PC1 and little influence on the PC2. In contrast, the *CPT1* gene was located on quadrant *a*, with positive scores for PC2.

**Table 4:** **Loadings for the first three principal components**^**1**^

**Variable**	**PC1**	**PC2**	**PC3**
TL	0.883	−0.259	0.021
14:0	0.579	−0.681	0.260
16:0	0.274	−0.740	0.526
16:1*c*9	0.778	−0.319	−0.415
18:0	−0.592	0.060	0.643
18:1*c*9	0.762	−0.062	−0.327
CLA	0.723	−0.006	−0.492
18:2*n*-6	−0.784	0.443	−0.051
*ACACA*	0.799	0.339	0.297
*CRAT*	0.798	0.348	0.211
*CPT1*	−0.402	0.464	0.092
*LPL*	0.836	0.364	0.120
*PPARA*	0.862	0.212	0.215
*PPARG*	0.926	0.091	0.188
*FABP4*	0.853	0.080	−0.152
*SCD*	0.845	0.341	0.077
*SREBF1*	0.816	0.270	0.234
Proportion of variance (%)	57.10	13.04	9.43
Cumulative variance (%)	57.10	70.14	79.57

^**1**^*PC *principal component, *TL *total lipids.

The PC1, which explains 57% of the variability, separated tissues (SAT *vs* muscle), with lipogenic genes and total lipids associated with SAT metabolism (quadrants *b* and *c*). The PC2, which explains 13% of the variability, separated palmitic (16:0) and myristic (14:0) fatty acids (quadrant *c*) from the remaining fatty acids. In quadrant *b*, a cluster was defined by *ACACA*, *CRAT*, *LPL*, *SCD*, *SREBF1* and *PPARA* genes. In addition, a cluster was formed by CLA, oleic acid (18:1*c*9) and the *FABP4* gene. The score plot depicted in Figure 3B shows the location of the LL muscle and SAT samples in the multivariate space of the first two PCs. These scores were clearly arranged in two clusters, corresponding to each tissue. In addition, for SAT there was some degree of separation between Alentejana (mostly located on quadrant *a*) and Barrosã bulls (mostly located on quadrant *d*), whereas for muscle the distinction between experimental groups was not so clear.

## Discussion

The deposition of fat and fatty acids in animal tissues has been ascribed to a complex regulation network of lipogenic genes, although the molecular mechanisms underlying these systems remain to be established. Considering that adipose tissue physiology is related to both meat quality and animal production efficiency, understanding the factors affecting the depot-specific fat accretion and metabolism in beef cattle is of paramount importance. The present study addressed these aspects based on an experimental trial with two genetically diverse bovine breeds with distinct maturity rates, Alentejana and Barrosã, fed diets with different silage to concentrate ratios (30/70% *vs.* 70/30%). Albeit phylogenetically distant [18], Alentejana and Barrosã breeds are, nevertheless, more genetically similar than the breeds used in previous studies addressing adipogenic gene expression differences, mainly based on the Japanese Black genotype [20-22]. Nonetheless, results reported here indicate that genetic background and, to a lesser extent diet composition, determine fat content and composition, pointing out to a differential fat partitioning between subcutaneous and intramuscular fat in Alentejana and Barrosã breeds.

In order to elucidate the molecular mechanisms involved in this physiological process, these breed- and diet-specific variations are explained here through the transcript levels of nine key lipid metabolism-related genes. The PCA showed a close relationship among all adipogenic and lipogenic genes, which separated distinctively from *CPT1* gene, involved in the β-oxidation pathway, but not *CRAT*. *PPARG* is a key activator of adipocyte differentiation and insulin sensitivity [23], inducing the transcription of many adipocyte genes encoding proteins and enzymes involved in the development and the maintenance of the adipocyte phenotype [24]. Surprisingly, levels of expression of *PPARG* were kept unchanged among experimental groups in the SAT samples, although Barrosã breed tended to have higher expression levels in the muscle than Alentejana bulls. It was expected that Barrosã breed, being a precocious breed, would had a greater proportion of adipocytes undergoing differentiation and, therefore, would had a greater relative expression of *PPARG* than Alentejana bulls. Although *PPARG* expression peaks during adipocyte differentiation, it is also expressed in mature adipocytes, but at lower levels [2]. Harper and Pethick [25] reported that the expression of *PPARG* decreases substantially as growth proceeds. Therefore, treatment differences may not have been detected if one group had adipocytes undergoing differentiation, whereas the other group may have greater number of mature adipocytes resulting in similar *PPARG* expression levels. Differences could, however, be detected at earlier stages of development. These results can be explained by the circumstance that subcutaneous fat develops earlier than intramuscular fat and, therefore, the tendency observed in the muscle was not detected in the SAT.

The *SREBF1* gene encodes a transcription factor involved in adipocyte differentiation [26] and in the biosynthesis of fatty acids [27] being, possibly, implicated in a mechanism that links adipogenesis and lipogenesis. In particular, the SREBF1c isoform appears to be primarily involved in regulating the expression of lipogenic and fatty acid-metabolizing enzymes. The transcription and activation of SREBF1c protein was proposed to be regulated by the degree of saturation of lipids [28]. Graugnard *et al*. [29] suggested that the expression of the *SREBF1* gene could be nutritionally regulated. Transcriptional regulation of *SREBF1* in most non-ruminant animals is sensitive to insulin, which under times of carbohydrate excess leads to stimulation of fatty acid synthesis and TAG deposition in adipose tissue [30]. In the present study, insulin levels were also the highest in low silage fed bulls [31], as were the expression levels of *SREBF1* in the SAT. Although the transcription factor *SREBF1* is considered to regulate *SCD* expression [32], its expression levels were not consistent with that of *SCD* in the muscle.

As pre-adipocytes differentiate into mature adipocytes, *LPL* is one of the first genes expressed due to its role in the *de novo* fatty acid synthesis [24]. A higher relative *LPL* mRNA expression in the SAT could indicate that Barrosã bulls had more adipocytes undergoing differentiation when compared to Alentejana bulls. Pickworth *et al*. [2] proposed that *LPL* may be a more definitive indicator of adipocyte differentiation than *PPARG.* Concerning the relationships between the fatty acids and *LPL* expression in SAT, the correlation analysis revealed close relationships between levels of *LPL* mRNA and 18:1*c*9, as well as *c*9,*t*11-CLA, among others. These results highlight the role of *LPL* in the control of TAG uptake and, consequently, on the fatty acid profile. However, the same pattern was not observed for the muscle and *LPL* showed no clear association with the main fatty acids. Taking into account that both *SCD* and *LPL* are expressed late in adipogenesis, increased gene expression would be consistent with more active adipocytes in SAT from Barrosã than in Alentejana bulls. Taken together, these results suggest that Barrosã bulls may have more differentiated adipocytes, which are capable of storing fat in the subcutaneous depot.

FABP4 protein is responsible for the transport of fatty acid outside the cell [33] and plays a role in lipolysis and fatty acid trafficking in different tissues [34-36]. Fat storage and metabolism within functional adipocytes are modulated by FABP4 [24], and thus levels of expression of this gene can be used as a marker of fully differentiated adipocytes [2]. In fact, its association with intramuscular fat content and backfat thickness have been reported by Jurie *et al*. [37]. Results herein presented for *FABP4* expression are concomitant with a higher fatty acids deposition in the SAT from Barrosã when compared to Alentejana bulls. The higher expression levels of *FABP4* in Barrosã bulls fed the low silage diet may be indicative of a higher level of adipogenic differentiation in the SAT of these animals. Several studies reported an association between bovine *FABP4* gene expression or protein activity and intramuscular fat content [8,26,37], as well as backfat depth [38]. Accordingly, in this study, the Barrosã carcasses had the highest intramuscular fat and carcass fatness scores (data not shown). Furthermore, levels of *FABP4* expression were moderately associated with total lipids contents in the muscle, as well as with the 18:1*c*9 proportions and the associated desaturation index. On the other hand, *FABP4* was associated with the main fatty acids in the SAT. These results reinforce the role of *FABP4* in intramuscular fat development and the SAT as the major site for lipid metabolism in ruminants.

ACACA is a key regulator of lipogenesis and the rate-controlling enzyme in adipose tissue of meat-producing animals [39]. The expression of the *ACACA* gene is highly inducible in the major lipogenic tissues [39] and the enzyme is nutritionally regulated [40,41]. In a study by Joseph *et al*. [41], it was found that oleic acid had an inhibitory effect on the expression of lipogenic genes in the SAT. In line with this, data herein presented revealed that, in SAT, *ACACA* expression levels tended to be the highest in Barrosã bulls fed the low silage diet (lowest oleic acid content). However, in the muscle, the expression levels tended to be lower in the high silage fed animals. These results suggested that the effect of dietary fatty acid composition is not only influenced by the genetic background but also by the fat depot location. In addition, *ACACA* mRNA levels were shown to have a positive association with total lipid content in the muscle samples, thus adding evidence to the importance of this enzyme to intramuscular fat deposition in ruminants.

Dietary *n*-3 and *n*-6 PUFA have been shown to inhibit *de novo* lipogenesis in dairy cattle [42]. Accordingly, our results showed a tendency for higher *ACACA* expression levels in the muscle from the low silage- in comparison to the high silage-fed bulls. Underwood *et al*. [43] reported a positive relationship between ACACA enzyme activity and intramuscular fat, which is consistent with our findings. In the present study, total lipids in the muscle were positively related to the expression of *ACACA*. Although this gene codifies for an enzyme which catalyses the formation of SFA, there was a positive association between *ACACA* expression levels and TFA. This suggests that an increase in TFA biosynthesis, that is, in desaturase activity, is a major factor in intramuscular (but not SAT) deposition in ruminants. In addition, *CPT1B* tended to show lower expression levels in the muscle from Alentejana, indicating higher fatty acid oxidation in the Barrosã breed.

Results concerning *SCD* gene expression, considered a marker of mature adipocytes [29], support the concept of a higher degree of maturity of the SAT adipocytes from the Barrosã than the Alentejana bulls, as observed for the *FABP4* gene. The fatty acid composition of SAT mirrors the action of the SCD protein on substrates like stearic and palmitic acids. The correlation analysis between oleic acid proportions and *SCD* expression levels in the SAT was showed to be positive and significant. This result is in agreement with previous reports [44] that reported that increased SCD activity is, at least partially, responsible for an elevated oleic acid, the main MUFA, content in ruminant. Similarly, a significant correlation was also found between *c*9,*t*11-CLA percentage and *SCD* expression levels. Taniguchi *et al*. [22] reported a positive correlation between levels of *SCD* mRNA and MUFA proportion, which led to the conclusion that *SCD* expression might contribute to the differences in the SAT fatty acid composition between breeds. The variations observed in the fatty acid classes in the SAT and muscle samples were mostly due to breed. High MUFA proportions in SAT have been reported [12] and might be related to an elevated SCD activity. Overall, our results support the concept of a central role for *SCD* in adipose tissue fatty acid synthesis. Genes encoding lipogenic enzymes responsible for the *de novo* SFA synthesis and MUFA production were down-regulated in Alentejana breed when compared to Barrosã bulls. This is in agreement with changes in tissue fatty acid composition, in which 16:0 concentration, the end product of *de novo* SFA synthesis, and *c*9,*t*11-CLA, a product of delta-9 desaturation, were reduced in the SAT of Alentejana bulls when compared to that of Barrosã animals.

In contrast to what was observed in SAT, the expression levels of the *SCD* gene in the muscle were similar among the four experimental groups. Several authors reported that *SCD* mRNA [26] or protein expression levels [45] in muscle do not reflect intramuscular fat levels. The results herein reported are consistent with those of the previous studies. In addition, an association between *SCD* mRNA levels and MUFA contents in bovine SAT has been reported in several works [46-48]. Nonetheless, the same association has not been reported for muscle [45,47,49], which could point to a depot-specific regulation mechanism of *SCD* gene expression and/or enzyme activity. Finally, it should also be noted that there was a high individual variation in the *SCD* mRNA levels. Therefore, despite changes in relative gene expression mirroring the changes in MUFA proportion, the correlation analysis failed to establish a significant association between both. The lack of association between *c*9,*t*11-CLA and *SCD* expression is in agreement with data reported by both Ward *et al*. [42] and Bartoň *et al*. [50]. Positive correlations, however, were found between the levels of *SCD* gene expression and total lipids, as well as TFA, both of which increase as fattening proceeds [51]. It should however be taken into account that conclusions drawn from single-point observations may lead to the erroneous assumption that *SCD* was not affected and/or had a crucial role in intramuscular fat synthesis. In that regard, Graugnard *et al*. [29] suggested that responses to high starch diets might not necessarily lead to increased adipogenesis. Moreover, these authors reported with some surprise that animals fed a low starch/high fibre diet during the growing phase showed increases in expression of lipogenic genes *PPARG*, *FABP4*, and *SCD* during the finishing phase.

The *PPARA* gene induces the expression of the fatty acid β-oxidation genes [50,52]. The tendency for a higher expression of *PPARA* in the SAT from Barrosã bulls fed the low silage diet in comparison to the remaining experimental groups is concomitant with a more intense β-oxidation in the former breed. In addition, PPARA has been shown to induce the expression of delta-5 and delta-6 desaturase genes [53]. However, results from the present study revealed no clear association between the *PPARA *mRNA levels and the fatty acid composition of SAT.

Carnitine acyltransferases catalyse the exchange of acyl groups between carnitine and coenzyme A (CoA) [54]. These enzymes include CRAT and carnitine palmitoyltransferases (CPTs) [54]. The CPTs transesterify medium and long chain fatty acyl chains, whereas CRAT transesterifies short-chain acyl chains [55]. High *CRAT* gene transcription levels may be indicative of an elevated number of differentiating cells during growth [54]. The higher *CRAT* transcription levels found in the Barrosã bulls are in agreement with the higher lipid accumulation in the Barrosã SAT, in comparison to the Alentejana bulls. In contrast, the lack of a significant variation in their relative expression levels of both genes’ in muscle samples is in agreement with the similar expression levels observed for the genes indicative of terminal adipocyte differentiation (*SCD*, *LPL* and *PPARG*). In addition, the close association between *CRAT* and the genes involved in lipogenesis reinforces the relationship between lipogenesis and β-oxidation, thus being indicative of high lipid turnover in those animals with high lipid deposition.

If in SAT there was a clear effect of breed in fatty acid deposition, with no influence of diet composition, in the LL muscle the response to dietary silage levels depended on animals’ genetic background. In previous studies [56,57], diets differing in starch contents (high starch *vs*. low starch), which is also the case of the present study, resulted in higher intramuscular fat in animals fed high starch diets. The interaction between breed and diet found suggests that the concept that high concentrate diets increase beef intramuscular [13] may be determined by breed and/or maturity. The differential response to diet composition between Alentejana and Barrosã bulls suggests distinct ruminal biohydrogenation patterns, as indicated by the intramuscular fatty acids contents, as well as the increased 18:2n-6 metabolic availability in Alentejana bulls fed the low silage diet. A differential regulation of fatty acid metabolism in distinct fat depots could explain the fact that no similar response was observed in SAT. Indeed, various fat depots have been reported to differ markedly in lipogenic activity [58,59]. An increase in the SFA content is to be expected whenever the concentrate proportion in the diet is increased. Feed silage level had no effect on total SFA of both tissues. However, in the SAT stearic acid was higher in high silage than in low silage fed bulls, and also higher in Alentejana when compared to Barrosã breed, but no such effects were found in the LL muscle. Similarly, both total and individual MUFA were not responsive to dietary silage level in the SAT, whereas in the LL muscle a tendency was found for the low silage diet to promote higher percentages of these fatty acids. Both PUFA and TFA were influenced by breed and diet, but the response to breed and diet factors differed between tissues. The BCFA were the only fatty acids showing similar response to diet, given that they originate ruminal activity and suffer no further modifications until being deposited in tissues. Taken together, these results suggest a differential regulation of fatty acid metabolism between tissues, possibly resulting from the contrasting roles of intramuscular and subcutaneous fat depots. In addition, the data also indicate differences in genetic background reflected in the response to diet composition, both in the gene expression of adipogenic and lipogenic factors and fatty acid composition of tissues.

The PCA confirmed the tissues contrasting features regarding lipid metabolism and fatty acid composition, showing a clear separation between the muscle and the SAT, as was previously shown by the analysis of variance. In addition, this statistical approach showed that there is less variability in muscle fatty acid composition and gene expression when compared to the SAT, as depicted by the plot of component scores. Furthermore, the PCA indicates that the expression levels of most adipogenic and lipogenic genes, along with linoleic acid, are the variables with the most discriminant power between tissues.

## Conclusions

The results herein presented suggest that, at 18 months old, Barrosã bulls might have more differentiated adipocytes and lipids deposited in SAT than Alentejana animals. Moreover, both lipogenesis (*SCD* and *LPL*) and β-oxidation (*CRAT*) related genes had higher levels of mRNA in the SAT from Barrosã animals when compared to Alentejana bulls. These data indicate a higher storage/removal ratio of TAG and a greater dynamics of lipid turnover in the SAT of Barrosã breed relative to Alentejana bulls.

The fatty acid deposition in the SAT is mainly influenced by dietary silage level, whereas the effect of breed is mostly associated with the expression level of the transcription factor *SREBF1*. Combined effects of breed and diet were obtained for the *de novo* fatty acid synthesis (*ACACA*) and fatty acid transport in adipocytes (*FABP4*) related genes, and the transcription factor *PPARA* mRNA levels.

In contrast to SAT, only a slight breed effect was obtained for muscle, with the expression levels of *PPARG* and *CPT1B* showing a tendency to be higher in Barrosã bulls. However, the low silage diet, relative to the high silage diet, increased the levels of *FABP4* and *ACACA* mRNA and tended to decrease *LPL* expression in the muscle.

Taken together, the results herein presented show that lipid metabolism in SAT is more sensitive to breed than muscle, whereas lipid metabolism in the latter tissue appears to be mostly diet-dependent. The differential gene expression patterns in SAT and muscle are likely responsible for the fatty acid partitioning between both tissues, thus reinforcing the prevailing role of SAT over intramuscular fat in the *de novo* fatty acid synthesis. These findings provide evidence for breed- and tissue-specific variations in fatty acid content and composition of beef cattle, which can be explained, at least in part, by the expression of key adipogenic and lipogenic genes involved in lipid metabolism. This insight into the molecular mechanisms underlying fat deposition in bovine SAT and muscle in different breed may contribute to the development of diet-based strategies to improve competitiveness of beef industry in order to satisfy consumers’ expectations.

## Methods

### Animals and experimental diets

All experimental procedures involving animals were reviewed by the Ethics Commission of CIISA/FMV and approved by the Animal Care Committee of the National Veterinary Authority (Direcção-Geral de Veterinária, Lisbon, Portugal), following the appropriated European Union guidelines (Directive 86/609/EEC). This trial was conducted at the facilities of Unidade de Produção Animal, L-INIA, INRB (Fonte Boa, Vale de Santarém, Portugal), from January to November 2009. Forty young bulls from Alentejana (n = 20) and Barrosã (n = 20), were assigned to high or low forage based diets (four experimental groups of 10 animals each). One Alentejana bull from the high silage fed group was later removed from the trial due to a limp. Diets were composed of 30/70% (low silage) and 70/30% (high silage) of maize silage and concentrate, respectively. The detailed proximate and fatty acid composition of the experimental diets has been published in a previous paper [60]. Briefly, crude fat and starch were higher in the low silage (31.7 and 376 g/kg DM, respectively) than in the high silage diet (28.7 and 285 g/kg DM, respectively). Conversely, the high silage diet had higher crude fibre and NDF contents (198 and 403 g/kg DM, respectively) in comparison to the low silage diet (150 and 321 g/kg DM, respectively). The low silage diet had lower palmitic (20.2 *vs*. 24.1%) and stearic acid (5.1 *vs*. 9.4 %) percentages than the high silage diet, while the latter showed higher proportions of 20:0 (6.5 *vs*. 3.7%), 18:2*n*-6 (43.9 *vs*. 40.9%) and 18:3*n*-3 (9.2 *vs*. 6.0%). Animals were housed in eight adjacent pens, two pens per breed and diet. The initial age was 331 ± 32 days for Alentejana bulls (average weight of 266 ± 45.8 kg) and 267 ± 10 days for Barrosã bulls (average weight of 213 ± 16.3 kg). All animals were slaughtered at 18 months of age, which is the commercial slaughter age for bulls in Portugal. Slaughters were performed at the INRB experimental abattoir by exsanguination, after stunning with a cartridge-fired captive bolt stunner.

Results concerning productive performance were described previously [61]. Briefly, Alentejana bulls fed the high silage diet had an average weight of 622 ± 17.7 kg at slaughter, whereas the average weight for those fed the low silage diet was 636 ± 29.7 kg. Regarding Barrosã bulls, the weight at slaughter was 457 ± 8.88 kg for those fed the high silage diet and 497 ± 23.0 kg for bulls fed the low silage diet. Dry matter intake and average daily gain were higher in Alentejana when compared to Barrosã bulls, although feed efficiency was similar across experimental groups. Fatness scores were higher in Barrosã than in Alentejana bulls. Moreover, the low silage fed animals had higher fatness scores than those fed the high silage diet.

### Sample collection

Immediately after slaughter, samples of *longissimus lumborum* muscle and SAT for gene expression analysis were collected from the right side of carcass at the 5th lumbar vertebra level, rinsed with sterile RNAse-free cold water solution, cut into small pieces (thickness of ~0.3 cm), stabilised in RNA Later solution (Qiagen, Hilden, Germany) and subsequently stored at −80°C. A second sample of SAT was vacuum-packed and stored at −20°C, until lipid extraction and determination of fatty acid composition.

Carcasses were suspended from the Achilles tendon, chilled at 10°C for 24 hours and aged during 8 days at 2°C. The left half carcass was subsequently separated into commercial joints. The LL muscle samples (*ca*. 200 g) were collected, trimmed of connective and adipose tissues before being blended in a food processor, vacuum packed and stored at −20°C until lipid analysis.

### Total lipid content and fatty acid composition

SAT and *longissimus lumborum* muscle samples were lyophilised (−60°C and 2.0 hPa), maintained at −20°C (SAT) or dissecated at room temperature (muscle) and analysed within two weeks. Total lipids were extracted by the method of Folch *et al.*[62], using dichloromethane and methanol (2:1 v/v) instead of chloroform and methanol (2:1 v/v), as modified by Carlson [63].

Fatty acids were then converted to methyl esters as described by Raes *et al*. [64], using sodium methoxide in anhydrous methanol (0.5 mol/l) for 30 min, followed by hydrochloric acid in methanol (1:1 v/v) for 10 min at 50°C. Fatty acid methyl esters (FAME) were extracted twice with 3 ml of *n*-hexane and pooled extracts were evaporated at 35°C, under a stream of nitrogen, until a final volume of 2 ml. The resulting FAME were then analysed by GC using a fused-silica capillary column (CP-Sil 88; 100 m × 0.25 mm i.d., 0.20 mm film thickness; Chrompack, Varian Inc., Walnut Creek, CA, USA), equipped with a flame ionisation detector, as described by Bessa *et al.*[65]. The quantification of FAME used nonadecanoic acid (19:0) as the internal standard, added to lipids prior to saponification and methylation. The same FAME solution was used for the analysis of both fatty acid composition and *c*9,*t*11-CLA, enabling the direct comparison of quantitative data and eliminating differences in sample preparation. CLA isomers were individually separated by triple silver-ion columns in series (ChromSpher 5 Lipids; 250 mm × 4.6 mm i.d., 5 μm particle size; Chrompack, Bridgewater, NJ, USA), using a high performance liquid chromatography (HPLC) system (Agilent 1100 Series, Agilent Technologies Inc., Palo Alto, CA, USA) equipped with an autosampler and a diode array detector adjusted to 233 nm, according to the procedure previously reported [66]. Fatty acid composition was expressed as g/100 g of total fatty acid content, assuming a direct relationship between peak area and fatty acid methyl ester weight.

### Total RNA extraction

Frozen tissue samples were homogenized with an Ultra-Turrax® homogenizer (IKA-Labortechnik, Staufen, Germany). For SAT samples, total RNA was extracted using RNeasy Lipid Tissue Mini Kit(Qiagen Inc, Hilden, Germany) according to the manufacturer’s protocol. Total RNA was extracted from muscle samples using Trizol reagent (Invitrogen, Carlsbad, CA, USA) and purified with the RNeasy Mini Kit (Qiagen Inc), according to the manufacturer’s protocol. To exclude possible amplification of contaminating genomic DNA, an additional step of DNase digestion was performed with the RNase-free DNase Set (Qiagen Inc.), incubating samples with DNase for 15 min at room temperature. Total RNA extracts were immediately analyzed for quantity (OD260nm) and purity (OD260 nm/OD280 nm) (NanoDrop ND-2000 c, Peqlab GmbH, Erlangen, Germany). RNA aliquots were stored at −80°C and until further analysis.

### Synthesis of complementary DNA

Single-stranded cDNA was synthesised using the High Capacity cDNA Reverse Transcription Kit (Applied Biosystems, Foster City, CA, USA) following the manufacturer's protocol. Each 20 μl RT reaction contained 250 ng (SAT) or 600 ng (muscle) of RNA template, 50 nM random RT Primer, 1 × RT buffer, 0.25 mM of each dNTPs, 3.33 U/μl multiscribe reverse transcriptase and 0.25 U/μl RNase inhibitor, at temperatures of 25°C for 10 min, 37°C for 120 min, and 85°C for 5 min cDNA aliquots were stored at −20°C.

### Primer design and housekeeping gene stability evaluation

Primer Express software (Applied Biosystems, Foster City, CA, USA) was used to design primers fixing the amplicon length to 65–150 bp (Table 5). When possible, primers were designed to fall across exon–exon junctions. Primers were aligned against publicly available databases using BLASTN at the National Center of Biotechnology Information. Prior to RT-qPCR the various sets of gene-specific primers were tested using a conventional PCR and run in a 2.5% agarose gel stained with ethidium bromide. Only those primers that presented a single band at the expected size in the gel, and thus no primer-dimer formation, were used for RT-qPCR. The accuracy of primer pairs was also evaluated by the presence of a unique peak during the dissociation step at the end of RT-qPCR. A set of five candidate housekeeping genes (HKG) was evaluated using geNorm and NormFinder, following the procedures described by Vandesompele *et al.*[67] and Andersen *et al.*[68], respectively. Therefore, target gene expression levels were normalised against the expression levels of the HKG, ribosomal protein large P0 (*RPLP0)* and peptidylprolyl isomerase B (*PPIB)* for SAT and muscle, respectively.

**Table 5:** **Primer pairs sequences for quantitative real-time PCR**^**1-4**^

**Gene symbol**	**Full gene name**	**Acc. number **^**1**^	**Primer pairs (5’-3’)**^**2**^	**Exons spanned**	**Product size (bp)**
*ACACA*	Acetyl-CoA carboxylase alpha	NM_174224	F: ttc acg tgg cct ggg tag a	40-41	142
			R: ttg tac ctg gat tct cct tca tct t		
*CPT1B*	Carnitine palmitoyltransferase 1B	NM_001034349	F: gcg act cca gtg gga cat tc	12-13	114
			R: aaa ggc agg aac tgg aag ca		
*CRAT*	Carnitine O-acetyltransferase	NM_001075587	F: ggc cca ccg agc cta cac	12-13	138
			R: atg gca atg gcg tag gag gt		
*FABP4*	Fatty acid-binding protein 4	NM_174314	F: tgg atg ata aga tgg tgc tgg a	3-4	114
			R: atg gag ttc gat gca aac gtc		
*LPL*	Lipoprotein lipase	NM_001075120	F: act gtg gct gag agc gag aac	7-8	98
			R: tct cca ata tcc acc tcc gtg ta		
*PPARA*	Peroxisome proliferator-activated receptor alpha	NM_001034036.1	F: agt gcc ttt cag ttg gga tgt c	2-3	125
			R: cgc ggt ttc gga atc ttc ta		
*PPARG*	Peroxisome proliferator-activated receptor gamma	NM_181024	F: tgt ctc ata atg cca tca ggt ttg	4-5	66
			R: tct ccg cta aca gct tct cct t		
*SCD*	Stearoyl-CoA desaturase	NM_173959	F: cca tca acc ccc gag aga at	5-6	76
			R: aag gtg tgg tgg tag ttg tgg aa		
*SREBF1*	Sterol regulatory element binding transcription factor 1	NM_001113302	F: agc ctg gca atg tgt gag aag	13-14	115
			R: caa gga gca ggt cac aca gga		
*PPIB*^3^	Peptidylprolyl isomerase B	NM_174152	F: tcc gtc ttc ttc ctg ctg ttg	1-2	98
			R: cca att cgc agg tca aag tac		
*RPLP0*^4^	Ribosomal protein P0	NM_001012682	F: gca ttc ccg ctt cct gg	5-6	109
			R: gcg ctt gta ccc att gat ga		

^§^Published in [69].

^1^Entrez Gene, National Center for Biotechnology Information (NCBI).

^2^F: forward primer and R: reverse primer.

^3^Housekeeping gene for muscle.

^4^Housekeeping gene for subcutaneous adipose tissue.

### Real time quantitative polymerase chain reaction

The RT-qPCR was performed with the StepOne Plus™ Real-Time PCR System, using the Power SYBR® Green master mix (both Applied Biosystems, Foster City, CA, USA). Reaction mixes of 6.25 μL Power SYBR Green master mix (Applied Biosystems, Foster City, CA, USA), 1 μL of forward and reverse primers (160 nM) and 1 μL of diluted cDNA (1:10) template were pipetted into MicroAmp™ optical 96-Well reaction plates and sealed with optical caps (Applied Biosystems, Foster City, CA, USA). After an initial denaturation at 95°C for 10 min, a thermocycling program of 15 s at 95°C, 60 s at 60°C and 15 s at 95°C was applied (40 cycles). Total fluorescence data and dynamic well factors were continuously collected to generate background-subtracted amplification curves (StepOne™ Software v2.2.2, Applied Biosystems, Foster City, CA, USA). PCR analysis of cDNA samples was performed in duplicate, using no-transcription and no-template samples as controls. The specificity of the PCR amplification was confirmed by melting curve analysis and agarose gel electrophoresis of PCR products.

### Data processing

The PCR efficiency was calculated for each primer set using the StepOnePlus PCR System software (Applied Biosystems), by amplifying 5-fold serial dilutions of pooled cDNA and run in triplicate. The efficiency curves were used to assess accuracy, linearity and efficiency of the PCR reaction. Efficiency (E) was calculated as E [%] = (10-1/slope of standard curve - 1) × 100. All primer sets exhibited an efficiency ranged between 85 and 110% and correlation coefficients were higher than 0.990.

The relative expression (RE) levels were calculated as a variation of the Livak method [70], corrected for variation in amplification efficiency (E = 10^-1/slope^), as shown in Equation 1.

(1) $$\mathrm{RE}=E_{\text{endogenous}}\left(\mathrm{CT},\text{edogenous}\right)/\;E_{\text{target}}\left(\mathrm{CT},\text{target}\right)$$

### Statistical analysis

Statistical analyses were carried out with the Statistical Analysis Systems software package, version 9.2, (SAS Institute, Cary, NC, USA). All statistical analyses were performed based on a 2 × 2 factorial arrangement of breed (Alentejana and Barrosã purebreds), diet (high and low silage diets) and their respective interaction. The variances were tested for heteroscedasticity and, for most parameters, variance was found to be heterogeneous. Therefore, subsequent data analysis was performed in order to account for heterogeneous variance. The general Satterthwaite approximation was computed in a mixed-effects regression model (PROC MIXED), with breed, diet and their interaction as fixed effects.

The slaughter weight, total lipids content in the muscle and SAT were tested as covariates, but only total lipids was retained. Whenever the use of a covariate was necessary, the structure of the covariate model was determined according to the procedures described by Milliken & Johnson [71], ranging from a simple slope model to individual slopes for each diet × breed combinations. Given that significant differences in covariate ranges were intrinsically associated to each breed, each variable was adjusted and compared with the mean covariate value of each breed [71]. When significant effects were detected, least square means (LSMEANS) were determined using the LSMEANS option, with no correction for multiple comparisons. Differences were declared significant at *P* < 0.05 and tendencies discussed at *P* < 0.10.

Pearson correlation coefficients were calculated using the CORR procedure of SAS. Whenever necessary, adjusted variables to the intramuscular fat or the amount of subcutaneous adipose tissue in the leg joint were used to compute Pearson correlations, for the muscle and SAT samples respectively.

The relationships between cellularity and fatty acid composition in both depots were assessed by the principal component analysis (PCA), using the PRINCOMP procedure of SAS. The PCA was applied to a data set of 78 samples and 17 variables to reduce the dimensionality of the data set and to describe the variability of data. The PCA was used to examine the relationship between the fatty acid composition and relative gene expression levels, enabling not only plots of the relationship between the variables but also attempting to explain those relationships. The analysis was based on the correlation matrix and the principal components which explained at least 5% for the total variance and had eigenvalues were greater than one were retained.

## Abbreviations

ACACA: Acetyl-CoA carboxylase alpha; BCFA: Branched chain fatty acids; CRAT: Carnitine O-acetyltransferase; CLA: Conjugated linoleic acid; FABP4: Fatty acid binding protein 4; FAME: Fatty acid methyl esters; HKG: Housekeeping gene; HS: High silage; LL: Longissimus lumborum; LPL: *Lipoprotein lipase*; LS: Low silage; MUFA: Monounsaturated fatty acids; PPARA: Peroxisome proliferator-activated receptor alpha; PPARG: Peroxisome proliferator-activated receptor gamma; PPIB: Peptidylprolyl isomerase B; PUFA: Polyunsaturated fatty acids; RPLP0: Ribosomal protein P0; SAT: Subcutaneous adipose tissue; SFA: Saturated fatty acids; SCD: Stearoyl-CoA desaturase; SREBF1: Sterol regulatory element binding transcription factor 1; TAG: Triacylglycerols; TFA: *Trans* fatty acids.

## Competing interests

There are no conflicts of interest.

## Authors’ contributions

ASHC and VMRP performed the tissue sampling and laboratory work. ASHC was responsible for the statistical analysis. ASHC, VMRP, CMGAF and JAMP were responsible for interpretation of the results and preparation of the manuscript. JAMP was responsible for the design of the study. All authors read and approved the findings of the study.
